# Supplementary material for: Targeted and whole-genome sequencing reveal a north-south divide in P. falciparum drug resistance markers and genetic structure in Mozambique
Source: Commun Biol. 2023 Jun 8;6:619. doi: 10.1038/s42003-023-04997-7 (PMC10250372; doi:10.1038/s42003-023-04997-7)
Supplement: Supplementary file 2 — Supplementary Information [file 42003_2023_4997_MOESM2_ESM.docx]

**SUPPLEMENTARY INFORMATION**

**Supplementary information for the paper: Targeted and whole-genome sequencing reveal a north-south divide in *P. falciparum* drug resistance markers and genetic structure in Mozambique**

**Supplementary Table 1.** Sampling source of *P. falciparum* positive samples successfully genotyped for at least one molecular marker of resistance, whole genome sequences available, and samples with microhaplotype coverage higher than 50%.

**Supplementary Table 2.** Districts and Provinces in Mozambique where *P. falciparum* isolates successfully genotyped were collected.

**Supplementary Table 3.** Number of *P. falciparum* samples from which molecular markers of antimalarial resistance were obtained.

**Supplementary Table 4.** Molecular markers of *P. falciparum* antimalarial resistance observed at frequencies below 5% in 2015 and 2018 in 7 provinces from Mozambique.

wt, wild-type; mut, mutants; mix; mixed genotypes

**Supplementary Table 5.** Frequency of *P. falciparum* isolates carrying mixed genotypes in molecular markers of antimalarial resistance.

**Supplementary Table 6.** Frequency of *P. falciparum* isolates carrying mutations in in *pfdhfr, pfdhps and pfmdr1* by province.

Samples with a mixed genotype in codons 51, 59, 108 of *pfdhfr* and 437 or 540 in *pfdhps* were excluded. *pfdhfr* and *pfdhps* haplotypes were built as a combination of mutations at these codons. *pfdhfr* double mutants were defined as any combination of two mutations out of the three possible.*pfdhps*single mutants were defined as the presence of either*pfdhps-*437 or -540. P values indicate the statistical significance of the difference in frequencies between provinces.

wt, wild-type; mut, mutant

**Supplementary Table 7.** Frequency of *P. falciparum* isolates carrying mutations in *pfdhfr*, *pfdhps* and *pfmdr1* by year.

Samples with a mixed genotype in codons *pfdhfr*-51, -59 or -108, and *pfdhps-*437 or -540 were excluded. *pfdhfr* and *pfdhps* haplotypes were built as a combination of mutations at these codons. *pfdhfr* double mutants were defined as any combination of two mutations out of the three possible.*pfdhps*single mutants were defined as the presence of either*pfdhps-*437 or -540. P values indicates the statistical significance of the difference in frequencies between years.

wt, wild-type; mut, mutant

**Supplementary Table 8.** Multivariable logistic regression model, with presence or absence of a mutation as response and temporal and spatial predictors.

|  | **Region** | | | |  | **Period** | | | |
| --- | --- | --- | --- | --- | --- | --- | --- | --- | --- |
|  |  | **OR** | **(95%CI)** | **p** |  |  | **OR** | **(95%CI)** | **p** |
| **Monoclonal (n=1090)** | | |  |  |  |  |  |  |  |
|  | **North** | 1 |  | **0.013** |  | **2015** | 1 |  | 0.464 |
|  | **Central** | 1.29 | (0.87; 1.90) |  |  | **2018** | 0.88 | (0.63; 1.23) |  |
|  | **South** | 1.62 | (1.16; 2.26) |  |  |  |  |  |  |
| ***dhfr* 51 (n=1638)** | |  |  |  |  |  |  |  |  |
|  | **North** | 1 |  | **0.033** |  | **2015** | 1 |  | **0.009** |
|  | **Central** | 1.37 | (0.63; 2.98) |  |  | **2018** | 2.29 | (1.23; 4.29) |  |
|  | **South** | 2.67 | (1.25; 5.69) |  |  |  |  |  |  |
| ***dhfr* 59 (n=1625)** | |  |  |  |  |  |  |  |  |
|  | **North** | 1 |  | **<0.001** |  | **2015** | 1 |  | **0.002** |
|  | **Central** | 3.38 | (1.35; 8.45) |  |  | **2018** | 3.4 | (1.59; 7.29) |  |
|  | **South** | 7.66 | (2.93;20.04) |  |  |  |  |  |  |
| ***dhfr* 108 (n=1649)** | | |  |  |  |  |  |  |  |
|  | **North** | 1 |  | **0.005** |  | **2015** | 1 |  | **0.004** |
|  | **Central** | 3.1 | (0.92;10.48) |  |  | **2018** | 5.06 | (1.67;15.34) |  |
|  | **South** | 11.38 | (2.39;54.25) |  |  |  |  |  |  |
| ***dhfr* pure (n=1600)** | | |  |  |  |  |  |  |  |
|  | **North** | 1 |  | **0.006** |  | **2015** | 1 |  | **0.002** |
|  | **Central** | 1.53 | (0.76; 3.06) |  |  | **2018** | 2.46 | (1.40; 4.33) |  |
|  | **South** | 2.98 | (1.51; 5.89) |  |  |  |  |  |  |
| ***dhps* 436 (n=1539)** | | |  |  |  |  |  |  |  |
|  | **North** | 1 |  | **<0.001** |  | **2015** | 1 |  | 0.683 |
|  | **Central** | 0.09 | (0.04; 0.19) |  |  | **2018** | 0.87 | (0.44; 1.71) |  |
|  | **South** | 0.01 | (0.00; 0.04) |  |  |  |  |  |  |
| ***dhps* 437 (n=1439)** | | |  |  |  |  |  |  |  |
|  | **North** | 1 |  | **<0.001** |  | **2015** | 1 |  | **0.004** |
|  | **Central** | 3.28 | (2.18; 4.95) |  |  | **2018** | 1.78 | (1.20; 2.63) |  |
|  | **South** | 17.55 | (10.73;28.72) |  |  |  |  |  |  |
| ***dhps* 540 (n=1404)** | | |  |  |  |  |  |  |  |
|  | **North** | 1 |  | **<0.001** |  | **2015** | 1 |  | **0.003** |
|  | **Central** | 2.94 | (1.96; 4.41) |  |  | **2018** | 1.75 | (1.20; 2.55) |  |
|  | **South** | 12.92 | (8.22;20.32) |  |  |  |  |  |  |
| ***dhps* pure (n=1377)** | | |  |  |  |  |  |  |  |
|  | **North** | 1 |  | **<0.001** |  | **2015** | 1 |  | **0.009** |
|  | **Central** | 3.22 | (2.12; 4.89) |  |  | **2018** | 1.69 | (1.14; 2.50) |  |
|  | **South** | 15.03 | (9.32;24.24) |  |  |  |  |  |  |
| **quintuple pure (n=1330)** | | |  |  |  |  |  |  |  |
|  | **North** | 1 |  | **<0.001** |  | **2015** | 1 |  | **0.007** |
|  | **Central** | 2.77 | (1.85; 4.14) |  |  | **2018** | 1.67 | (1.15; 2.42) |  |
|  | **South** | 11.27 | (7.33;17.33) |  |  |  |  |  |  |
| ***mdr1* 184 (n=1171)** | | |  |  |  |  |  |  |  |
|  | **North** | 1 |  | 0.291 |  | **2015** | 1 |  | 0.354 |
|  | **Central** | 0.78 | (0.54; 1.11) |  |  | **2018** | 1.14 | (0.87; 1.50) |  |
|  | **South** | 0.93 | (0.68; 1.29) |  |  |  |  |  |  |

**Supplementary Table 9.** Frequency of *P. falciparum* isolates carrying mutations in codon 436 of *dhps*, in combination with other mutations in *pfdhfr* and *pfdhps*.

Samples with a mix genotype in any of the codons 51, 59, 108 of *pfdhfr* and 437 or 540 in *pfdhps* were excluded. P values indicates the statistical significance of the difference in frequencies.

**Supplementary Table 10**. Confusion Matrix for Random Forest classification at the regional (North-Central-South) level.

Microhaplotypes at loci with expected heterozygosity in the top 25% percentile were considered as predictors of the classification model. Random Forest was run with ntree = 2500 and the OOB error rate = 24.89%.

|  | **North** | **Central** | **South** | **Classification error** |
| --- | --- | --- | --- | --- |
| **North** | 98 | 66 | 34 | 0.5051 |
| **Central** | 49 | 12 | 57 | 0.4690 |
| **South** | 10 | 55 | 600 | 0.0977 |

**Supplementary Table 11.** Estimated genetic complexity of infection by region and year.

**Supplementary Table 12.** Expected heterozygosity (H_e_) values at 16 microhaplotype loci within a 50kb region around *pfdhps* in *P. falciparum* isolates from Cabo Delgado.

Haplotypes were constructed after excluding mixed genotypes in *pfdhps* codons 436/437/540. Sub-populations WT/WT/WT + MUT/WT/WT were merged for the analysis. The statistical significance of the difference of the H_e_ values between the groups was calculated using a permutation test, by randomly shuffling the sub-population labels of the samples 1000 times and obtaining a null distribution for the difference of the H_e_.

| **Microhaplotype** | **Gene** | **WT/WT/WT & MUT/WT/WT**  N=51 | **WT/MUT/MUT**  N=92 | **p value** |
| --- | --- | --- | --- | --- |
| **Pf3D7_08_v3-522797-522858** | *pfabc1* | 0.246 | 0.221 | 0.737 |
| **Pf3D7_08_v3-527371-527432** | *pfppm5* | 0.410 | 0.412 | 0.962 |
| **Pf3D7_08_v3-532468-532604** | *pf0810400* | 0.355 | 0.178 | 0.052 |
| **Pf3D7_08_v3-534632-534693** | *pfppm7* | 0.150 | 0.087 | 0.344 |
| **Pf3D7_08_v3-536195-536274** | *pfppm7* | 0.340 | 0.288 | 0.498 |
| **Pf3D7_08_v3-542446-542571** | *pfdbp1* | 0.682 | 0.533 | **0.006** |
| **Pf3D7_08_v3-543445-543506** | *pfdbp1* | 0.212 | 0.084 | 0.102 |
| **Pf3D7_08_v3-548133-548222** | *pfdhps* | 0.482 | 0.022 | **<0.001** |
| **Pf3D7_08_v3-548770-548831** | *pfdhps* | 0.511 | 0.104 | **<0.001** |
| **Pf3D7_08_v3-549650-549715** | *pfdhps* | 0.703 | 0 | **<0.001** |
| **Pf3D7_08_v3-549962-550023** | *pfdhps* | 0.079 | 0 | 0.304 |
| **Pf3D7_08_v3-556150-556203** | *pfcul1* | 0.393 | 0.166 | **0.005** |
| **Pf3D7_08_v3-557281-557382** | *pfcul1* | 0.292 | 0.257 | 0.649 |
| **Pf3D7_08_v3-559505-559566** | *pfemc1* | 0.402 | 0.044 | **<0.001** |
| **Pf3D7_08_v3-562482-562647** | *Intergenic* | 0.156 | 0.516 | **<0.001** |
| **Pf3D7_08_v3-565050-565130** | *pfcaf1* | 0.289 | 0.099 | 0.038 |

**Supplementary Figure 1.** Frequency of *P. falciparum* isolates carrying mutations in *codon 184 of pfmdr1.*

The error bars represent a 95% Confidence Interval (CI) for the population proportion.


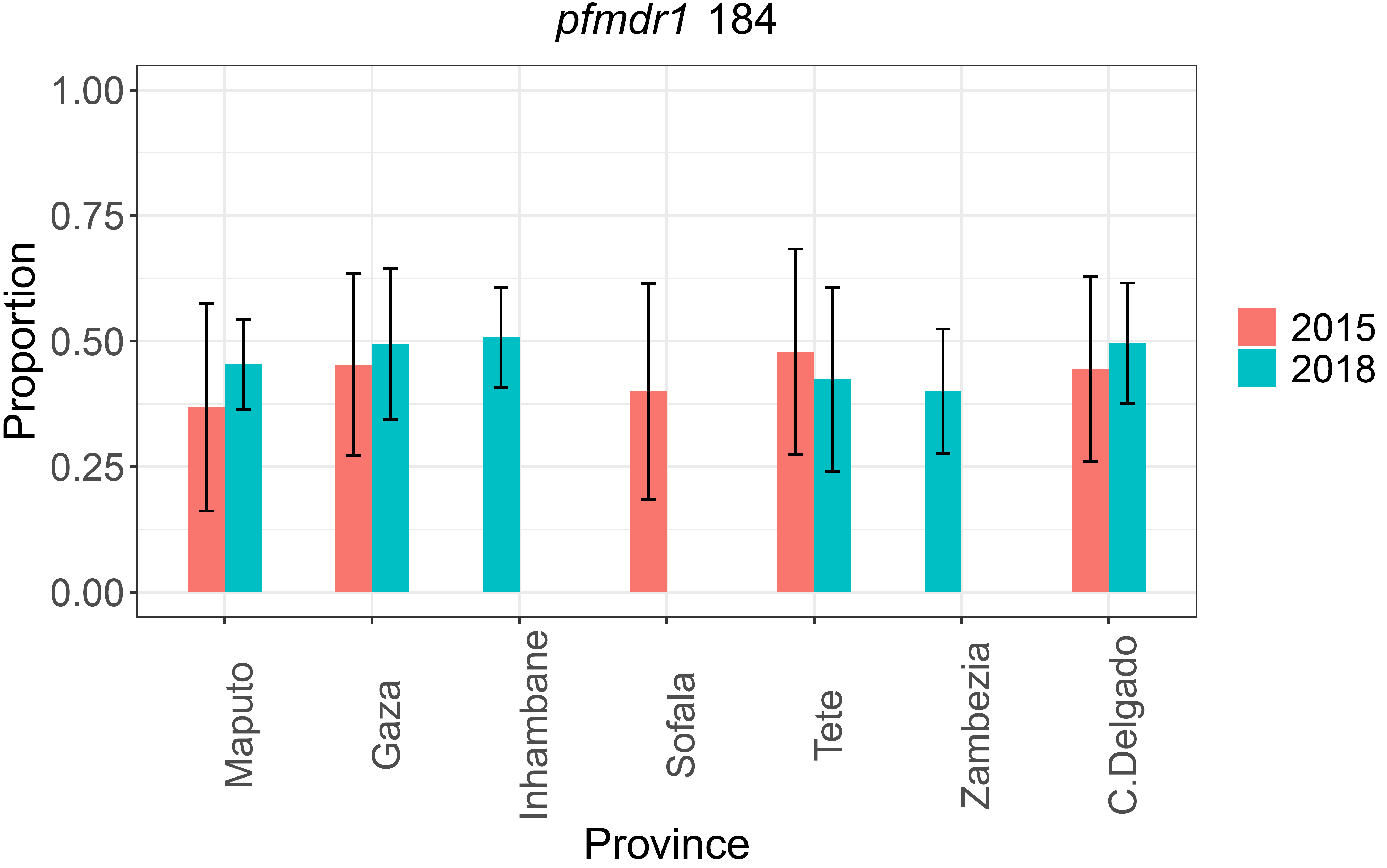


**Supplementary Figure 2.** Frequency of *P. falciparum* isolates carrying mutations in *pfdhfr* (codons 51,59,108) and *pfdhps* (codons 436, 437, 540) in 2015 and 2018 in 7 provinces from Mozambique.

Frequencies were calculated after excluding mixed genotypes. Data from Sofala was only available for 2015, and from Inhambane and Zambezia for 2018. The error bars represent a 95% Confidence Interval (CI) for the population proportion.


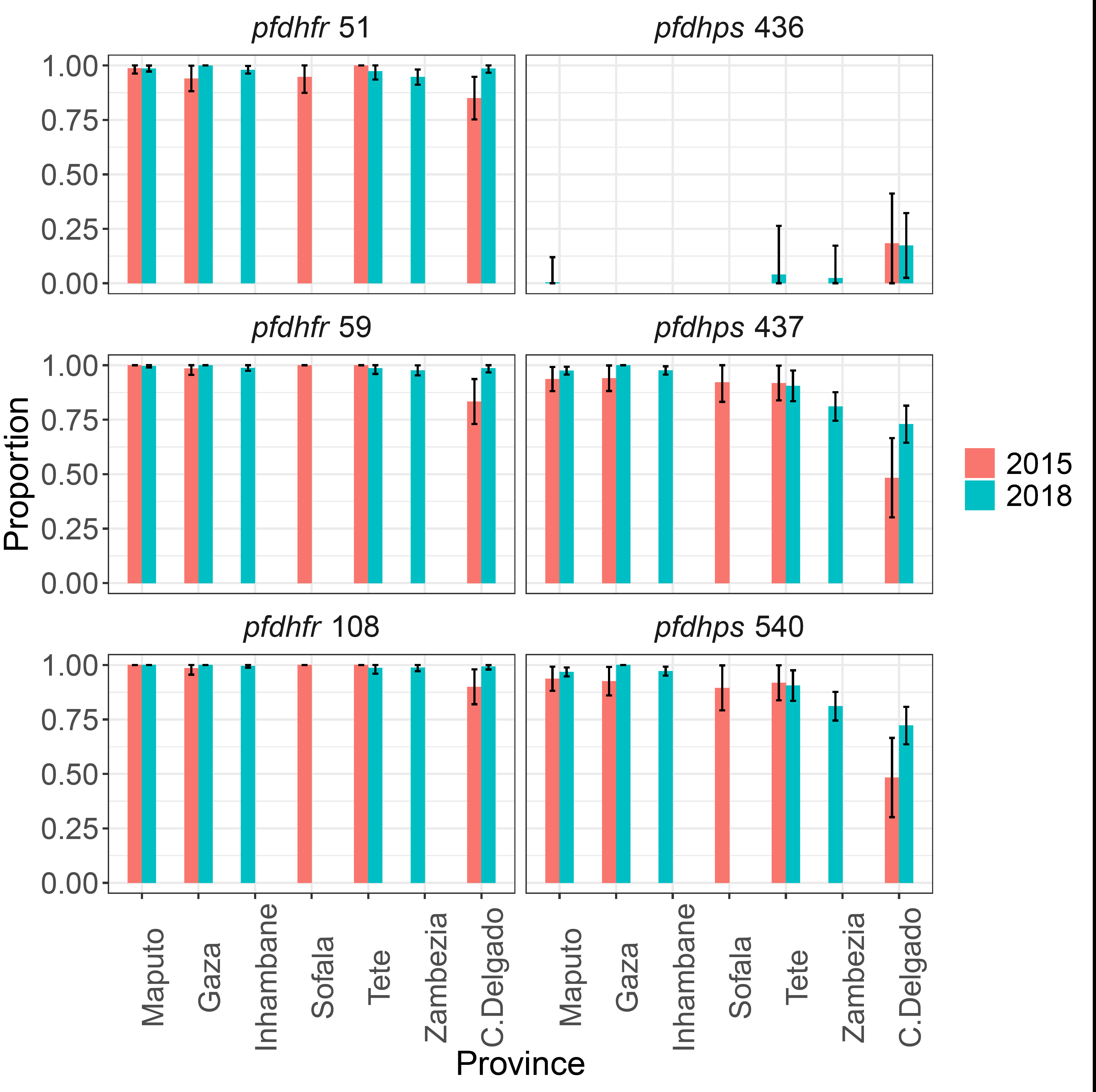


**Supplementary Figure 3.** Microhaplotypes in *P. falciparum* samples included in the study.

**A**) Percentage of microhaplotype loci (N=8722) present for samples from 2015 and 2018 (N=1438). Samples which had greater than 50% of the microhaplotype loci missing (N=349) were excluded from subsequent analyses. **B**) Microhaplotypes, sorted by importance, which contribute to the geographic (North-Central-South) classification model. 155 microhaplotypes had a mean decrease in accuracy greater than the inflection point (3.1; red dashed line) of the distribution of mean decrease in accuracy.


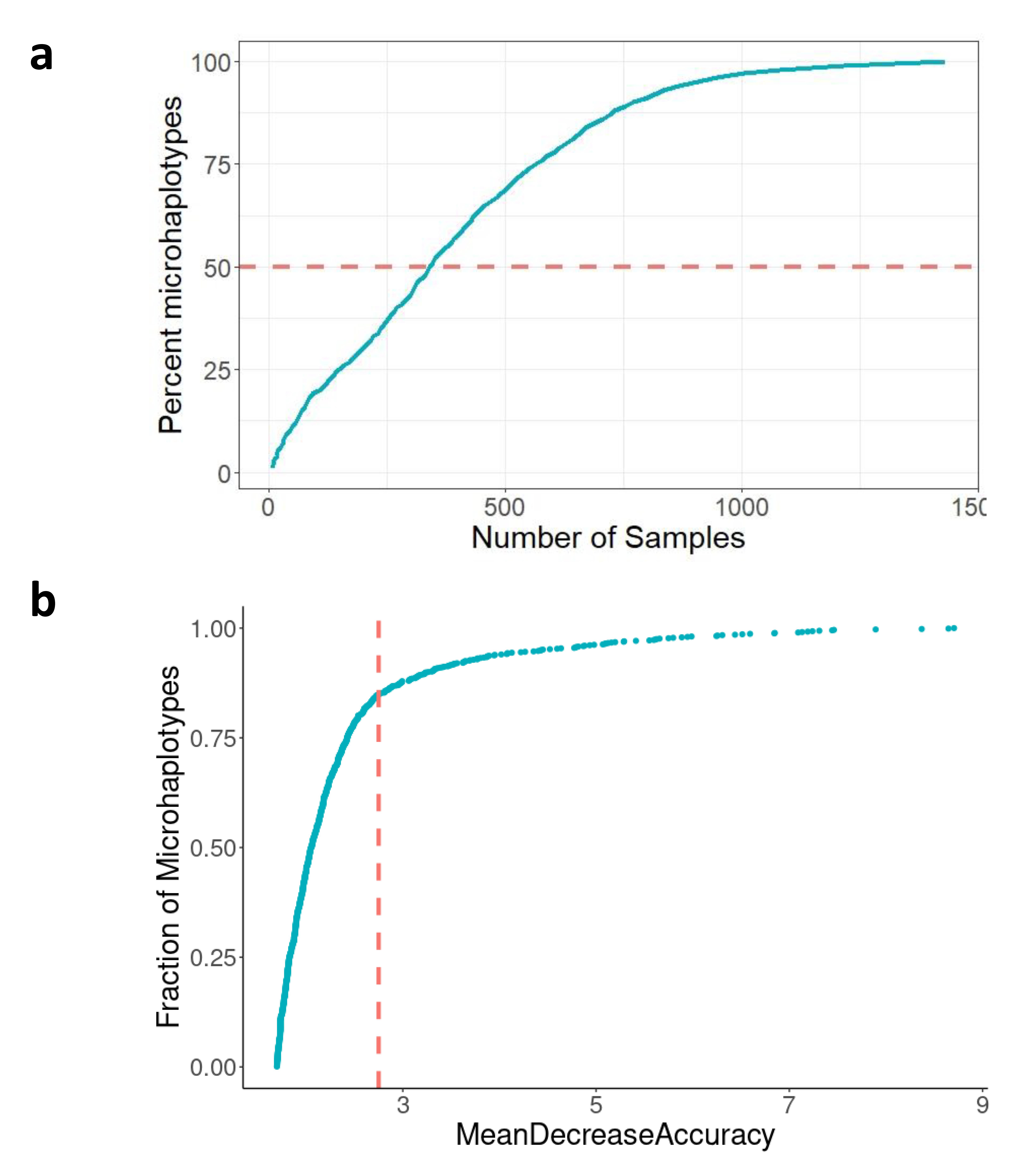


**Supplementary Figure 4.** Population structure by geography.

Principal coordinates analysis (PCoA) of the raw data, considering microhaplotypes at loci with expected heterozygosity in the top 25% percentile (left figure in each panel), and considering the top microhaplotypes important for each Random Forest classification (right figure in each panel). Microhaplotypes at loci with expected heterozygosity in the top 25% percentile were considered as predictors of the classification model. Random Forest was run with ntree = 2500. Sample size (2015 and 2018 together): C. Delgado: 198, Zambezia: 131, Sofala: 6, Tete: 89, Gaza: 166, Inhambane: 230, and Maputo: 269 (total: 1089). For the province level classification, samples from Tete and Sofala were combined together to reduce sample imbalance. Regional assignment of samples: North: C.Delgado; Central: Sofala, Tete and Zambezia; South: Gaza, Inhambane and Maputo. **A)** Random Forest classification at the province level. OOB error rate = 50.51%. **B)** Random Forest classification at the regional (North-South) level. OOB error rate = 8%. **C)** Random Forest classification at the regional (Central-South) level. OOB error rate = 15.26%. **D)** Random Forest classification at the regional (North-Central) level. OOB error rate = 36.79%


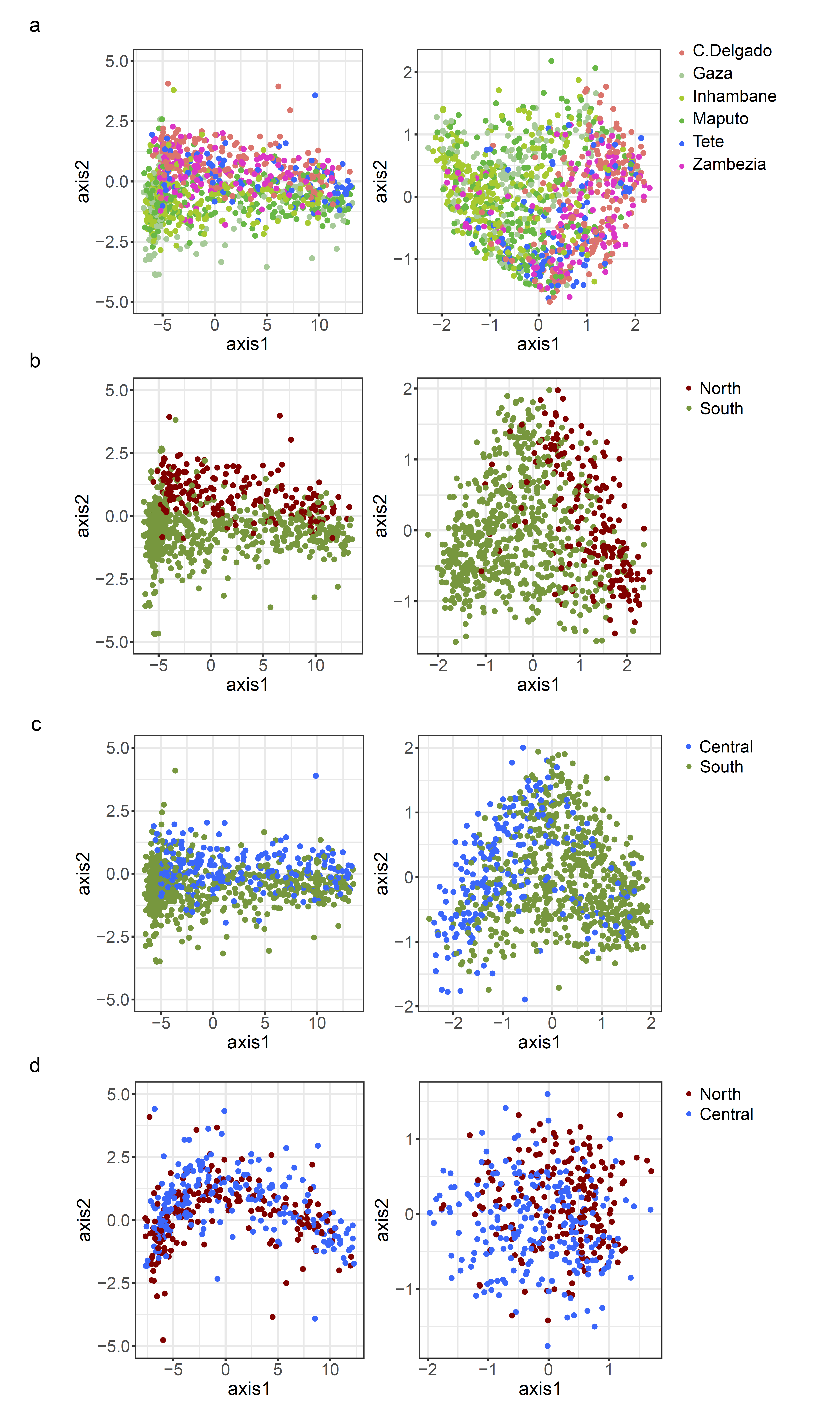


**Supplementary Figure 5.** Genetic diversity around *pfdhps* locus.

The expected heterozygosity at microhaplotypes within the 50kb region around *pfdhps* of parasite isolates from Cabo Delgado collected in 2015 and 2018. Wild-type in 436, 437 and 540 (WT/WT/WT; n=20) and mutant in 436, and wild-type in 437 and 540 (MUT/WT/WT; n=31) were combined and compared to wild-type in 436, and mutant in 437 and 540 (WT/MUT/MUT; n=92) population. The error bars indicate ± standard error (SE).

For loci falling within the gene *pfdhps* (chr 8: 547896-551057), this difference was particularly pronounced, with three of the four microhaplotype loci having significant differences in H_e_ values between the two groups. These four loci are: Pf3D7_08_v3-548133-548222 (*pfdhps* codons 1-7, 5 microhaplotypes), Pf3D7_08_v3-548770-548831 (*pfdhps* codons 133-152, 3 microhaplotypes), Pf3D7_08_v3-549650-549715 (*pfdhps* codons 426-446, 6 microhaplotypes) and Pf3D7_08_v3-549962-550023 (*pfdhps* codons 530-549, 3 microhaplotypes). The error bars indicate the standard error.


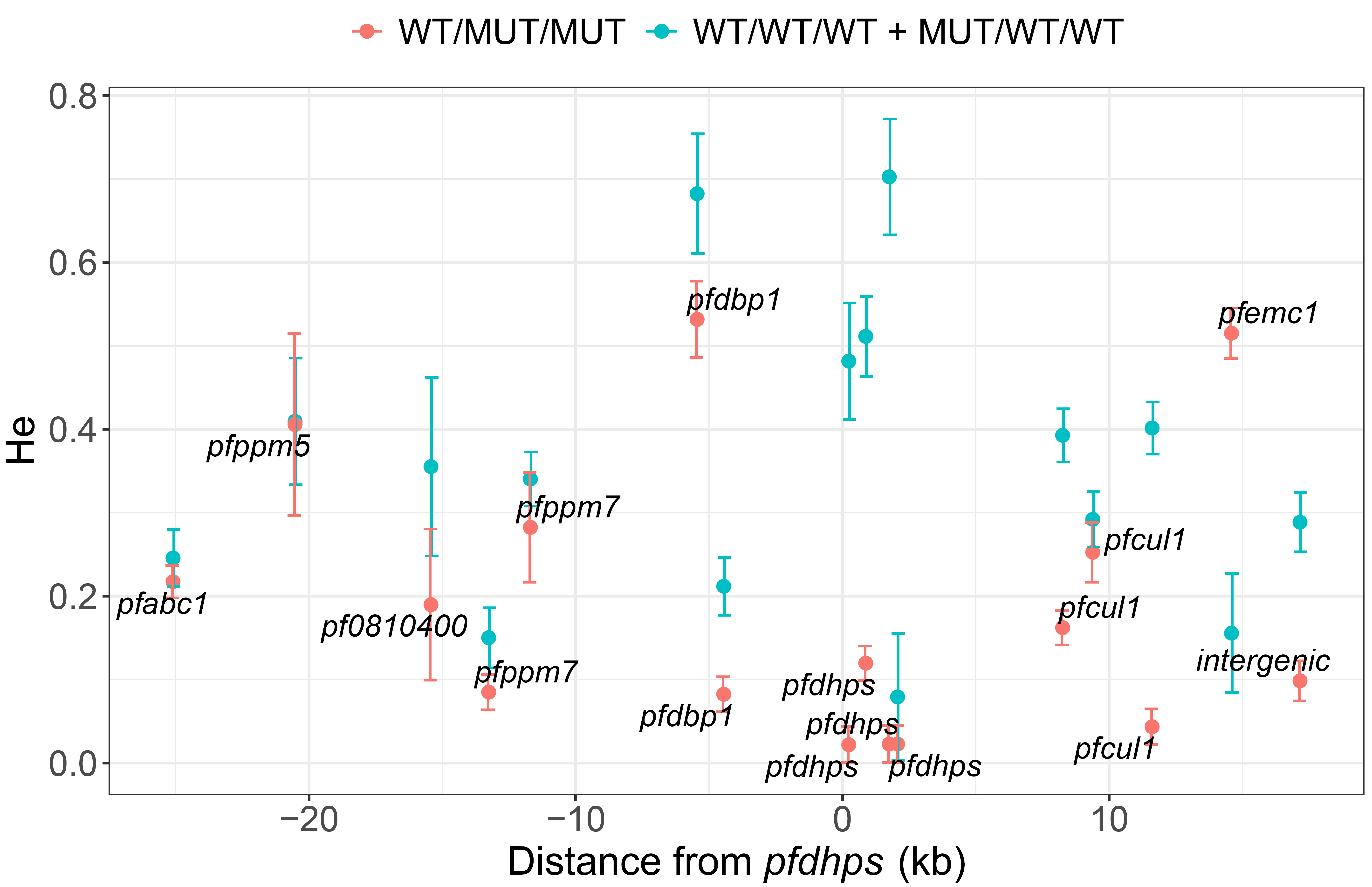

**Supplementary Figure 6.** Violin plots of inter-sub-population IBS values in Cabo Delgado (2015 and 2018).

Microhaplotypes in a 50 kb region around *pfdhps* were used to calculate the pairwise IBS between sub-populations: wild-type in codons 436, 437 and 540 (WT/WT/WT; n=20): median IBS=0.68, IQR=(0.62-0.74); mutant in codon 436, and wild-type in codons 437 and 540 (MUT/WT/WT; n=31): median IBS=0.73, IQR=(0.65-0.79); wild-type in codon 436, and mutant in codons 437 and 540 (WT/MUT/MUT; n=92): median IBS=0.88, IQR=[0.81-0.91]). Comparisons between groups were done using Kruskal-Wallis Test (p<0.001). The lower, middle and upper hinges of the rectangle correspond to the 25% quantile , median and 75% quantile of the distribution
