# Supplementary material for: Targeted and whole-genome sequencing reveal a north-south divide in P. falciparum drug resistance markers and genetic structure in Mozambique
Source: Commun Biol. 2023 Jun 8;6:619. doi: 10.1038/s42003-023-04997-7 (PMC10250372; doi:10.1038/s42003-023-04997-7)
Supplement: Supplementary file 5 — Reporting Summary [file 42003_2023_4997_MOESM5_ESM.pdf]

nature portfolio

Corresponding author(s): Alfredo Mayor

Last updated by author(s): May 18, 2023

Reporting Summary

Nature Portfolio wishes to improve the reproducibility of the work that we publish. This form provides structure for consistency and transparency in reporting. For further information on Nature Portfolio policies, see our Editorial Policies and the Editorial Policy Checklist.

Statistics

For all statistical analyses, confirm that the following items are present in the figure legend, table legend, main text, or Methods section.

n/a	Confirmed	
☐	☒	The exact sample size (*n*) for each experimental group/condition, given as a discrete number and unit of measurement
☐	☒	A statement on whether measurements were taken from distinct samples or whether the same sample was measured repeatedly
☐	☒	The statistical test(s) used AND whether they are one- or two-sided<br>*Only common tests should be described solely by name; describe more complex techniques in the Methods section.*
☐	☒	A description of all covariates tested
☐	☒	A description of any assumptions or corrections, such as tests of normality and adjustment for multiple comparisons
☐	☒	A full description of the statistical parameters including central tendency (e.g. means) or other basic estimates (e.g. regression coefficient) AND variation (e.g. standard deviation) or associated estimates of uncertainty (e.g. confidence intervals)
☐	☒	For null hypothesis testing, the test statistic (e.g. *F*, *t*, *r*) with confidence intervals, effect sizes, degrees of freedom and *P* value noted<br>*Give P values as exact values whenever suitable.*
☒	☐	For Bayesian analysis, information on the choice of priors and Markov chain Monte Carlo settings
☒	☐	For hierarchical and complex designs, identification of the appropriate level for tests and full reporting of outcomes
☒	☐	Estimates of effect sizes (e.g. Cohen's *d*, Pearson's *r*), indicating how they were calculated

Our web collection on statistics for biologists contains articles on many of the points above.

Software and code

Policy information about availability of computer code

Data collection	REDCap
Data analysis	R

For manuscripts utilizing custom algorithms or software that are central to the research but not yet described in published literature, software must be made available to editors and reviewers. We strongly encourage code deposition in a community repository (e.g. GitHub). See the Nature Portfolio guidelines for submitting code & software for further information.

Data

Policy information about availability of data

All manuscripts must include a data availability statement. This statement should provide the following information, where applicable:

- Accession codes, unique identifiers, or web links for publicly available datasets
- A description of any restrictions on data availability
- For clinical datasets or third party data, please ensure that the statement adheres to our policy

The sequences have been deposited in the European Nucleotide Archive (ENA) under Project Name PRJEB2136 and the Sequence Read Archive (SRA) under BioProject ID PRJNA910151. Microhaplotype data used to assess the P. falciparum population structure by geography in Mozambique (Figure 3CD) are deposited in FigShare (https://figshare.com/s/1920d5bad8268218b480 and https://figshare.com/s/464a6825e09691aec654). A deidentified and restricted dataset can be provided by approved request after completion of a data use agreement by emailing to corresponding author.

Human research participants

Policy information about studies involving human research participants and Sex and Gender in Research.

Reporting on sex and gender	Both male and female individuals donated blood samples for genotyping of malaria parasites if infected. We did not perform sex-based analysis as the genetic profile of infecting parasites is not expected to be shaped by sex.
Population characteristics	The covariate-relevant population characteristic used in this study is area of residence in Mozambique (Province level).
Recruitment	Dried blood spots (DBS) that were evaluated represent a convenience sample obtained from individuals participating in different studies: six malaria observational studies and clinical trials conducted in 2015 and 2018. In 2018, two health facility survey studies recruited individuals attending outpatient services in Maputo, Zambézia, Cabo Delgado, Inhambane and Gaza (all ages). Samples from an additional two therapeutic efficacy studies included children less than 5 years of age with confirmed malaria (by rapid diagnostic test) in Cabo Delgado, Tete, Sofala and Gaza province in 2015 and Cabo Delgado, Tete, Zambézia and Inhambane in 2018). In the fifth study, all age individuals with a malaria positive RDT were identified through community-based cross-sectional surveys in Maputo Province (2015 and 2018), including a malaria elimination project area which collected samples from individuals participating in mass drug administration campaigns and reactive surveillance in Magude District. Finally, in the sixth study, pregnant women at first antenatal care (ANC) visit with a P. falciparum infection confirmed by quantitative real-time PCR (qPCR) were identified through ANC surveys conducted in Maputo Province (2018). Health facility-based sampling sites were district or subdistrict health centres or provincial hospitals, selected by the Centro de Investigação em Saúde de Manhiça (CISM) or National Malaria Control Programme (NMCP) according to their public health or research needs, whereas cross-sectional surveys were community-based and participants were randomly selected. Further information on sampling for each study is available in the associated publications provided as references in the manuscript.
Ethics oversight	All study protocols were approved by the Mozambican National Committee for Bioethics in Health.

Note that full information on the approval of the study protocol must also be provided in the manuscript.

Field-specific reporting

Please select the one below that is the best fit for your research. If you are not sure, read the appropriate sections before making your selection.

☒ Life sciences ☐ Behavioural & social sciences ☐ Ecological, evolutionary & environmental sciences

For a reference copy of the document with all sections, see nature.com/documents/nr-reporting-summary-flat.pdf

Life sciences study design

All studies must disclose on these points even when the disclosure is negative.

Sample size	A total of 2251 samples were collected in 2015 (n=724) and 2018 (n=1527) from 40 districts in seven provinces from Mozambique.
Data exclusions	Sequencing produced at least one SNP with a valid resistance genotype (among 11 genetic markers targeted) in 1784 (79%) samples (455 from 2015 and 1329 from 2018; 308 from North, 440 from Central and 1034 from South Mozambique). Whole genome sequences were obtained from a total of 1452 (64%) samples which passed quality filters.
Replication	Amplicon-based and whole genome sequencing was performed only once per sample by following procedures already published: SpotMalaria platform (https://www.malariagen.net/resources/amplicon-sequencing-toolkit/p-falciparum-amplicontoolkit-protocols; Jacob CG et al.; Genetic surveillance in the Greater Mekong Subregion and South Asia to support malaria control and elimination; eLife 2021;10:e62997 DOI: 10.7554/eLife.62997 and MalariaGen et al. An open dataset of Plasmodium falciparum genome variation in 7,000 worldwide samples. Wellcome Open Res 6, 42, 2021. doi:10.12688/wellcomeopenres.16168.2); and University of California, San Francisco (Tessema, S. K. et al. Sensitive, Highly Multiplexed Sequencing of Microhaplotypes From the Plasmodium falciparum Heterozygome. J Infect Dis 225, 1227-1237, 2022; doi:10.1093/infdis/jiaa527).
Randomization	No experimental groups were used in this study, except for the comparisson of genetic characteristics of malaria parasites based on pfdhps haplotypes (codons 51, 59 and 108 of dhfr and 436, 437 and 540 of dhps).
Blinding	Investigators were blinded to procedence and other characteristics of samples during the sequencing process.

Reporting for specific materials, systems and methods

We require information from authors about some types of materials, experimental systems and methods used in many studies. Here, indicate whether each material, system or method listed is relevant to your study. If you are not sure if a list item applies to your research, read the appropriate section before selecting a response.

Materials & experimental systems

n/a	Involved in the study
☒	☐ Antibodies
☒	☐ Eukaryotic cell lines
☒	☐ Palaeontology and archaeology
☒	☐ Animals and other organisms
☒	☐ Clinical data
☒	☐ Dual use research of concern

Methods

n/a	Involved in the study
☒	☐ ChIP-seq
☒	☐ Flow cytometry
☒	☐ MRI-based neuroimaging
